# Gender Differences of Schizophrenia Patients With and Without Depressive Symptoms in Clinical Characteristics

**DOI:** 10.3389/fpsyt.2021.792019

**Published:** 2022-01-13

**Authors:** Ruimei Liu, Xinyu Fang, Lingfang Yu, Dandan Wang, Zenan Wu, Chaoyue Guo, Xinyue Teng, Juanjuan Ren, Chen Zhang

**Affiliations:** ^1^Schizophrenia Program, Shanghai Mental Health Center, Shanghai Jiao Tong University School of Medicine, Shanghai, China; ^2^Biochemical Pharmacology Laboratory, Shanghai Mental Health Center, Shanghai Jiao Tong University School of Medicine, Shanghai, China; ^3^Nanjing Brain Hospital, Nanjing Medical University, Nanjing, China

**Keywords:** schizophrenia, depressive symptoms, psychiatric symptom, cognition, gender difference

## Abstract

**Objectives:** To investigate the differences in psychotic symptoms and cognitive function in schizophrenics with and without depression and to compare gender differences in the correlation between depressive symptoms and clinical characteristics in those patients.

**Methods:** A total of 190 schizophrenia patients and 200 healthy controls were recruited in the study. We used the Positive and Negative Symptom Scale (PANSS), the Calgary Depression Scale for Schizophrenia (CDSS) and the Repeatable Battery for the Assessment of Neuropsychological Status (RBANS) to evaluate the psychiatric symptoms, depressive symptoms and cognitive function, respectively. Patients with CDSS score ≥7 were divided into depression group, and CDSS < 7 was viewed as without depression.

**Results:** Patients with schizophrenia had lower total scores of RBANS and five subscale (immediate memory, visual span, verbal function, attention, and delayed memory) scores compared to healthy controls. In the case group, patients who concomitant with depression had higher PANSS scores (*P*s < 0.001) and lower RBANS (*P*s < 0.05) scores than those without depression. After gender stratification, PANSS total scores and subscale scores were significantly different between schizophrenics with and without depressive symptoms in both male and female groups (*P*s < 0.001). For cognitive function, there were significant differences in RBANS total score and subscale scores except attention between female patients with and without schizophrenia but not in male schizophrenia patients. Furthermore, the correlation analysis showed that the total CDSS score was positively correlated with PANSS score (*P* < 0.001) and RBANS score in male and female groups (male: *P* = 0.010, female: *P* = 0.001).

**Conclusion:** Our findings provided evidence supporting the gender differences in psychiatric symptoms and cognitive function between schizophrenia patients with and without depressive symptoms.

## Introduction

Depression is a common symptom in schizophrenia and can occur in any phase. It is one of the most frequent prodromal symptoms and may proceed before the onset of psychotic symptoms (1). Throughout the course of schizophrenia, depression predominates in the acute phase, with a prevalence frequency of 80% (2). When depressive symptoms are emerged without concomitant psychotic symptoms several months after an acute psychotic episode, it is termed as post-psychotic depression (3, 4). Although the occurrence of affective components has long been thought to predict a good prognosis in schizophrenia (5), ample evidence supports that the presence of depression in schizophrenia is associated with worse outcomes, a poorer occupational functioning, a greater risk of relapse, a higher frequency of rehospitalization, and an increased risk of suicide (2, 6, 7).

Evidence shows that the prevalence rates of depression in schizophrenia varies from 30 to 70% (8–10). The wide range difference in prevalence estimates could be attributed to cohort status, illness status (acute or chronic), and definition of depression. From a longitudinal point of view, the vast majority (up to 80%) of patients suffered at least one depressive episode in the early phase, and at least 30% of patients exhibited a major depressive episode in their lifetime (11, 12).

Additionally, the different assessment tools used in previous investigations is one of the most important factors contributing to the prevalence difference. As we know, the most commonly used scales to assess depressive symptoms are the Montgomery-Asberg Depression Rating Scale (MADRS) and the Hamilton Depression Rating Scale (HDRS) (13, 14), but these two scales are not suitable for schizophrenia patients owing to some semiology overlaps between negative symptoms and depression in schizophrenia. Hence, more tools are being developed. The Calgary Depression Scale for Schizophrenia (CDSS) is regarded as the best evaluation tool for depression in Chinese patients with schizophrenia which has the advantage of avoiding disturbance of negative symptoms and drug-induced extrapyramidal side effects. Our recent study used this tool and found that the rate of depressive symptoms in schizophrenia patients was 43.68%, which was similar in most previous studies used the same tool (15–17).

Moreover, the differences in gender ratio of schizophrenia patients included in previous studies may also account for the different rate of depressive symptoms. Substantial evidence has shown that many characteristics of schizophrenia are gender-specific, such as lifetime prevalence (18, 19), age of onset (20, 21), and comorbidities (22, 23). Numerous previous studies have found that schizophrenia patients with different gender also have different cognitive impairment characteristics (24, 25). Male schizophrenia patients tend to develop worse cognition than females on many domains, like reasoning, processing speed, visual learning, immediate memory and delayed memory. However, few reports focused on the gender differences of the comorbidity of depression in schizophrenia. Given that depressive symptoms are widespread in schizophrenia, it is necessary to take them into account when analyzing the clinical characteristics and cognitive function. We hypothesized that depressive symptoms may affect clinical characteristics and cognition in schizophrenia and with a gender specific. Therefore, we conducted this study was: (1) to investigate whether there were differences in psychotic symptoms and cognitive function of schizophrenics with and without depression; (2) to compare gender differences in the correlation between depressive symptoms and clinical characteristics.

## Methods

### Participants

Patients were recruited from the Shanghai Mental Health Center, and the inclusion criteria has already been published in the previous study (15, 26). In brief, patients were included if they met the following criteria: (1) diagnosis of schizophrenia according to Diagnostic and Statistical Manual of Mental Disorders, Fourth Edition (DSM-IV); (2) between 18 and 50 years old; (3) at least junior high school diploma; (4) no typical antipsychotics, mood stabilizer, and antidepressant was used within one month. The exclusion criteria were as follows: (1) organic mental disorders; (2) with comorbid substance abuse/dependence; (3) severe physical or neurological illnesses; (4) pregnant or breastfeeding. At the same time, 200 age and gender-matched healthy subjects were recruited as controls. All procedures had strictly been in accord with the Declaration of Helsinki and other relevant national and international regulations. We also obtained the written informed consent prior to any procedures related to this study being performed.

### Clinical Evaluation

We used the positive and negative symptom scale (PANSS) and Calgary Depression Scale for Schizophrenia (CDSS) to evaluate the psychiatric symptoms and depressive symptoms, respectively. There are nine structural items in the CDSS: depression, hopelessness, self-deprecation, guilty ideas of reference, pathologic guilt, morning depression, early awakening, suicide, and observed depressive symptoms (27). All items are scored on a scale of 0 ~ 3, and the cores were positively correlated with symptom severity. Patients were viewed as suffering notable depression by the total CDSS score of ≥7 (15, 28, 29). The RBANS includes five domains of neurocognition: immediate memory, visuospatial/constructional, attention, language, and delayed memory, which was used to evaluate the cognition function.

### Data Analysis

Statistical analysis in our study was run by SPSS 26.0. The independent-samples *T*-test was used to compare continuous variables between groups, and the chi-square test was used for categories data. Correlation analysis was used to explore the correlation between depressive symptoms and clinical features in patients with schizophrenia, and gender stratification was conducted. Age, body mass index (BMI), education level, age of onset, and total course of disease were potentially controlled as covariates. All statistical tests were conducted on two sides, and the significance level was *P* < 0.05.

## Results

### Comparisons Between Case and Control Groups

A total of 190 schizophrenia patients and 200 healthy controls were included in this study. There were no significant differences in age, sex, and education between the two groups (*P*s > 0.05). Schizophrenia patients showed higher BMI than the control group (*P* = 0.002). Most of the patients included suffered an antipsychotics-treated history; accordingly, the higher BMI could be partly due to weight gain induced by antipsychotics. The cognitive function between the two groups was statistically different (*P*s < 0.001). Schizophrenia patients got lower total RBANS scores and five subscale scores, including immediate memory, visuospatial/constructional, attention, language, and delayed memory. Table 1 shows the main sociodemographic and clinical characteristics between schizophrenia patients and the control group.

**Table 1:** The demographics and RBANS scores of the case and control groups.

	**Case**	**Control**	**t/X^**2**^**	***P***
	***n* = 190**	***n* = 200**		
Age (year)	34.35 ± 9.78	33.39 ± 8.83	*t* = 1.013	0.312
Sex (male/female)	89/101	90/110	*X*^2^ = 0.133	0.761
Education (year)	12.58 ± 3.42	12.93 ± 2.66	*t* = −1.121	0.263
BMI (kg/m^2^)	23.87 ± 3.86	22.74 ± 3.31	*t* = 3.102	0.002
RBANS	364.20 ± 67.73	462.48 ± 55.15	*t* = −15.666	<0.001
Immediate memory	64.15 ± 17.38	85.92 ± 16.43	*t* = −12.716	<0.001
Visuospatial	73.98 ± 15.61	82.97 ± 16.89	*t* = −5.447	<0.001
Language	73.33 ± 17.31	93.68 ± 14.98	*t* = −12.389	<0.001
Attention	88.82 ± 17.44	110.14 ± 16.40	*t* = −12.445	<0.001
Delayed memory	63.99 ± 19.74	89.68 ± 11.66	*t* = −15.552	<0.001

*BMI, Body mass index; RBANS, the Repeatable Battery for the Assessment of Neuropsychological Status*.

*Data were present in Mean ± SD or N*.

### Comparisons of Clinical Features Between Schizophrenia Patients With and Without Depression

Among 190 patients, 80 (42.1%) were determined to have depressive symptoms (CDSS score ≥ 7), 39 were male patients and 41 were female patients; 110 (57.9%) were viewed as no depressive symptoms (CDSS score < 7), 50 were male patients and 60 were female patients. There were no significances between the two groups in age, sex, BMI, years of education, age of onset, and total course of the illness (*P*s > 0.05).

We compared the total PANSS score and three subscale scores (positive score, negative score, and general psychopathology score), patients with depressive symptoms got higher total score and subgroup scores than patients who had no depressive symptoms (*P*s < 0.001). Cognitive measurements demonstrated that patients with depressive symptoms got lower scores than patients without depressive symptoms in total score and five subscale scores (*P*s < 0.05), as shown in Table 2.

**Table 2:** Comparisons between schizophrenia patients with and without depression.

	**With depression**	**Without depression**	**t**	***P***
	***n* = 80**	***n* = 110**		
Age (year)	35.42 ± 9.59	33.56 ± 9.88	1.298	0.196
Education (year)	12.66 ± 3.18	12.51 ± 3.59	0.296	0.768
BMI (kg/m^2^)	23.86 ± 3.75	23.88 ± 3.95	−0.029	0.977
Age of onset (year)	23.64 ± 6.88	23.64 ± 7.19	0.004	0.997
Illness course (month)	139.76 ± 101.54	123.67 ± 93.30	1.131	0.259
PANSS	71.29 ± 10.61	54.20 ± 10.06	4.554	<0.001
PANSS-P	16.15 ± 5.65	12.81 ± 4.46	7.744	<0.001
PANSS-N	19.44 ± 5.11	13.55 ± 5.25	9.015	<0.001
PANSS-G	35.36 ± 6.06	27.88 ± 5.33	1.298	<0.001
RBANS	343.44 ± 63.18	379.31 ± 67.20	−3.724	<0.001
Immediate memory	58.86 ± 15.52	68.00 ± 17.70	−3.701	<0.001
Visuospatial	69.71 ± 11.99	77.09 ± 17.18	−3.302	0.001
Language	69.63 ± 18.08	76.02 ± 16.29	−2.548	0.012
Attention	85.64 ± 18.24	91.12 ± 16.53	−2.161	0.032
Delayed memory	59.74 ± 18.15	67.07 ± 20.34	−2.615	0.011

*BMI, Body mass index; PANSS, the positive and negative symptom scale; RBANS, the Repeatable Battery for the Assessment of Neuropsychological Status*.

### Gender Differences in Schizophrenia Clinical Features Between Patients With or Without Depressive Symptoms

In the male group, patients with and without depression showed no differences in age, years of education, BMI, the total course of illness, family history, and drug-treated history (*P*s > 0.05). Female patients showed no differences in the above information between the two groups (*P*s > 0.05) except the total illness course; patients with depression suffered a more extended period of illness than those without depression (*P* = 0.015) (Table 3).

**Table 3:** Gender differences in schizophrenia clinical features between patient with and without depressive symptoms.

	**Male**		**t/X^**2**^**	***P***	**Female**		**t/X^**2**^**	***P***
	**With depression (*n* = 39)**	**Without depression (*n* = 50)**			**With depression (*n* = 41)**	**Without depression (*n* = 60)**		
Age (year)	35.00 ± 9.98	33.50 ± 9.72	0.714	0.477	35.83 ± 9.30	33.62 ± 10.10	1.116	0.267
Education (year)	12.49 ± 3.13	12.11 ± 3.26	0.552	0.583	12.83 ± 3.26	12.85 ± 3.84	−0.028	0.977
BMI (kg/m^2)^	23.94 ± 3.58	24.96 ± 3.73	−1.310	0.194	23.80 ± 3.95	23.00 ± 3.93	1.023	0.309
Age of onset (year)	25.11 ± 6.23	23.24 ± 7.82	1.220	0.226	22.25 ± 7.25	23.97 ± 6.66	−1.230	0.222
Illness course (month)	115.49 ± 98.35	133.15 ± 97.72	−0.844	0.401	162.85 ± 100.26	115.77 ± 89.50	2.472	0.015
Family history			0.029	0.865			0.103	0.749
No	31 (79.5%)	39 (78.0%)			31 (75.6%)	47 (78.3%)		
Yes	8 (20.5%)	11 (22.0%)			10 (24.4%)	13 (21.7%)		
Drug-treated history			1.595^a^	0.438			8.053^a^	0.012
Monotherapy	22 (56.4%)	34 (68.0%)			15 (36.6%)	39 (65.0%)		
Combination	14 (35.9%)	12 (24.0%)			24 (58.5%)	19 (31.7%)		
Drug-naive	3 (7.7%)	4 (8.0%)			2 (4.9%)	2 (3.3%)		
PANSS	69.23 ± 10.85	55.58 ± 10.02	6.149	0.000	73.24 ± 10.13	53.05 ± 10.03	9.897	<0.001
PANSS-P	15.46 ± 6.16	13.48 ± 4.56	1.682	0.097	16.80 ± 5.11	12.25 ± 4.34	4.822	<0.001
PANSS-N	18.82 ± 5.09	14.54 ± 5.96	3.581	0.001	20.02 ± 5.13	12.73 ± 4.46	7.591	<0.001
PANSS-G	34.69 ± 6.20	27.56 ± 5.25	5.872	0.000	36.00 ± 5.93	28.15 ± 5.42	6.879	<0.001
RBANS	342.58 ± 60.35	366.18 ± 63.48	−1.778	0.079	344.26 ± 66.49	390.25 ± 68.76	−3.345	0.001
Immediate memory	57.62 ± 14.41	63.76 ± 16.12	−1.867	0.065	60.03 ± 16.60	71.54 ± 18.31	−3.219	0.002
Visuospatial	71.00 ± 11.67	77.06 ± 17.11	−1.894	0.062	68.48 ± 12.30	77.12 ± 17.38	−2.923	0.004
Language	70.91 ± 16.94	74.65 ± 14.68	−1.112	0.269	68.41 ± 19.22	77.16 ± 17.55	−2.367	0.020
Attention	83.40 ± 19.68	88.42 ± 15.46	−1.347	0.181	87.77 ± 16.72	93.38 ± 17.18	−1.629	0.107
Delayed memory	59.83 ± 18.27	62.29 ± 19.76	−0.603	0.548	59.64 ± 18.26	71.06 ± 20.11	−2.907	0.005

*BMI, Body mass index; PANSS, the positive and negative symptom scale; RBANS, the Repeatable Battery for the Assessment of Neuropsychological Status*.

a *Fisher's exact test*.

In the psychological symptoms, female patients with depression showed more severe signs than patients without depression. They got significantly higher PANSS total score and all three subscale scores (*P*s < 0.001). In the male group, depressive patients also showed higher PANSS total scores and subscale scores, the slight difference was that there was no statistically significant difference in positive symptom score (*P* = 0.097). Cognition measurements demonstrated more gender differences. Patients with depression got lower RBANS total scores than patients without depression in both groups. However, statistical significance was found only in the female group (*P* = 0.001), but not in males (*P* = 0.079). Female patients with depression got lower scores in all five dimensions; the difference was statistically significant in all dimensions except attention (*P* = 0.107). However, this trend was slight in male patients. Although patients with depression got lower scores on all five dimensions, there was no significant difference in anyone subscale (*P*s > 0.05).

### The Gender Differences in the Correlation Between Depressive Symptoms and Clinical Features in Patients With Schizophrenia

Correlation analysis was used to explore the correlation between depressive symptoms and clinical characteristics (psychotic symptoms, cognitive function) in male and female schizophrenia patients, respectively. CDSS total score was viewed as a dependent variable, PANSS total score or RBANS total score as independent variable, and age, BMI, years of education, total course of the disease, and age of onset as covariates. Coefficient of correlation was shown in Table 4. The results showed a significant positive correlation between the depressive symptoms and psychotic symptoms (PANSS total score, male: *r* = 0.676, *P* < 0.001; female: *r* = 0.798, *P* < 0.001), cognitive function (RBANS total score, male: *r* = −0.208, *P* = 0.010; female: *r* = −0.327, *P* = 0.001) in both male and female patients. The female group showed a much stronger correlation in cognitive function, CDSS score had a statistically significant correlation with all RBANS subscales. The CDSS score had a statistically significant correlation with PANSS subscales in male patients, but the correlations with RBANS subscales were weak.

**Table 4:** Gender differences of correlations between CDSS total score and clinical variables in schizophrenia patients.

	**CDSS total scores**			
	**Male patients**		**Female patients**	
**Variable**	**r**	***P***	**r**	***P***
PANSS	0.676	<0.001	0.798	<0.001
PANSS-P	0.244	0.025	0.541	<0.001
PANSS-N	0.431	<0.001	0.661	<0.001
PANSS-G	0.681	<0.001	0.681	<0.001
RBANS	−0.280	0.010	−0.327	0.001
Immediate memory	−0.219	0.046	−0.281	0.006
Visuospatial skill	−0.201	0.067	−0.230	0.024
Language	−0.249	0.023	−0.201	0.049
Attention	−0.210	0.055	−0.204	0.046
Delayed memory	−0.139	0.206	−0.310	0.002

*PANSS, the Positive and Negative syndrome scale; CDSS, The Calgary Depression Scale for Schizophrenia; RBANS, the Repeatable Battery for the Assessment of Neuropsychological Status*.

## Discussion

It has been more than 100 years since Kraepelin used significant affective symptoms as one criterion for distinguishing manic-depressive illness from dementia praecox (30, 31). In recent decades, the Kraepelinian dichotomy has been increasingly challenged because emerging evidence revealed the overlap between psychosis and mood syndromes. Actually, depression is considered part of the core symptomatology of schizophrenia and is added as one dimension in the diagnosis of schizophrenia spectrum disorder (32). Our study found significant differences in clinical characteristics, including PANSS scores and RBANS scores, between schizophrenics with and without depressive symptoms. We also found that total CDSS score was strongly correlated with PANSS and RBANS scores.

Numerous studies have shown that depressive symptoms are associated with general psychopathology, positive symptoms, negative symptoms. Our study revealed that schizophrenia patients with depressive symptoms showed severer psychotic symptoms than those without depression, which was consistent with some previous findings (29, 33, 34). A large body of studies has specifically explored the correlation between depressive symptoms and psychiatric symptoms. Some studies suggested a clear correlation between depression and positive symptoms (29, 34). The results demonstrated that the increased depressive symptoms were generally linked to terrible positive symptoms (30). Compared with patients who co-morbidity of low depression, those co-morbidity of high depression exhibited significantly worse positive symptoms but not negative symptoms (35). However, some other researchers believed that depressive symptoms were secondary to negative symptoms (36). Evidence showed that CDSS scores were correlated most with negative symptom scores on the PANSS scale (20). Studies have shown that the alleviation of depressed mood may lead to reduced negative symptoms (37). Both negative and depressive symptoms are common symptoms in schizophrenia. How to distinguish them has always been the focus of attention. A recent systematic review focused on the phenomenological co-occurrence of negative symptoms and depression in schizophrenia, it proposed that pessimism, suicidal ideation, and low mood were specifically characteristic of depression, while alogia and blunted affect may be more specifically related to negative symptoms. Amotivation, anergia, anhedonia, avolition and emotional blunting occurred in both the negative and depressive domains (38).

Previous studies have reported extensive cognitive impairment in patients with schizophrenia and depression (39, 40). In this study, we found that patients with schizophrenia showed cognitive impairment in different dimensions. After gender stratification, a notable trend emerged in the female group that patients with depression got lower RBANS total score and subscale scores than those without depressive symptoms, namely patients with depression suffered severer cognitive impairment. Although male patients with depression also obtained lower scores in total RBANS and five subscales than patients without depression, the differences were not statistically significant. Cognitive impairment is the main predictor of prognosis and is closely correlated with poor social and occupational outcomes in schizophrenia. Studies have shown that people with schizophrenia exhibit progressive degenerative cognitive impairment. Accordingly, effective treatments are necessary to retard the progression of cognitive decline and prevent it from worsening.

Antipsychotics have long been considered the cornerstone of the treatment of schizophrenia. Monotherapy is recommended and its effectiveness is supported by solid evidence (41). Unfortunately, a single antipsychotic often leads to unsatisfactory effectiveness when multiple symptoms are occurred in an individual (42). Switching to or adding other antipsychotics is an alternative strategy, but little high-quality evidence is available for the effectiveness of these approaches (43, 44). From a point of reducing clinical symptoms, antidepressants are allowed to patients who do not respond to antipsychotic treatment. Results from a large cohort study showed that patients who initiating used an adjunctive antidepressant showed a reduced risk of emergency department visits and psychiatric hospitalization compared with switching to a new antipsychotic (43). Adjunctive antidepressant with medium- or high-dose antipsychotic lead to a lower risk of suicide and reduce overall mortality by 15~40% (45). However, a recent systematical analysis showed no statistically significant improvement in depression score with the combination of antidepressants (46). And another significant systematical study showed that primary outcomes (depressive and negative symptoms) indicated minor beneficial effects of adjunctive antidepressants (47). Experts recommend that antidepressants should be considered in patients whose depressive symptoms have not been improved in sufficient quantities and durations of new antipsychotics (48).

There are several limitations that should be noted. Firstly, our study is a cross-sectional study and no follow-up. Secondly, there is only a small sample size after gender stratification, which increases the occasional results, so the conclusion should be interpreted with caution. Finally, most patients included in the study were previously treated with antipsychotics and the use of antipsychotics may bias the results. Future longitudinal studies with large samples are needed to explore the gender differences of depressive symptoms in schizophrenia.

In conclusion, our results demonstrate that schizophrenia patients with depression show more severe psychotic symptoms and cognitive function than those without depression. The difference remains significant in female patients after gender stratification. Male patients only show differences in psychotic symptoms, but not in cognitive function. Total CDSS scores are significantly associated with PANSS and RBANS scores in both male and female patients.

## Data Availability Statement

The raw data supporting the conclusions of this article will be made available by the authors, without undue reservation.

## Ethics Statement

The studies involving human participants were reviewed and approved by the Institutional Review Boards of the Shanghai Mental Health Center. The patients/participants provided their written informed consent to participate in this study.

## Author Contributions

CZ, RL, and XF contributed to the overall design of the study. RL, XF, LY, DW, ZW, CG, XT, and JR got involved sample collection. RL and XF undertook the statistical analysis and interpretation of data. CZ and RL wrote the manuscript. All authors contributed to the article and approved the submitted version.

## Funding

This work was supported by National Key Research and Development Program of China [2018YFC1314302], National Natural Science Foundation of China [81471358 and 81771450], Western Medicine Guide Project of Shanghai Municipal Commission of Science and Technology [14411969000], Shanghai Municipal Education Commission-Gaofeng Clinical Medicine Grant Support [20152530], Shanghai Municipal Commission of Health and Family Planning Foundation [201540029], and Shanghai Municipal Commission of Health and Family Planning, Key Developing Disciplines [2015ZB0405].

## Conflict of Interest

The authors declare that the research was conducted in the absence of any commercial or financial relationships that could be construed as a potential conflict of interest.

## Publisher's Note

All claims expressed in this article are solely those of the authors and do not necessarily represent those of their affiliated organizations, or those of the publisher, the editors and the reviewers. Any product that may be evaluated in this article, or claim that may be made by its manufacturer, is not guaranteed or endorsed by the publisher.
